# Hospitals and the Conversion of Consols

**Published:** 1915-12-11

**Authors:** 


### HOSPITALS AND THE CONVERSION OF CONSOLS.

### Pros and Cons by a Secretary.

The figures relating to the exercise of the right of conversion of Consols and other Government issues into 4½% War Loan Stock have caused some little surprise that the liberal terms of conversion offered were not more fully taken advantage of.

It appears that out of a total of £920,000,000 stock carrying conversion rights, under £350,000,000 was converted. Included in these figures are £536,000,000 of Consols outstanding of which not more than £204,000,000 was converted. Consols held by Government Departments, £184,000,000, were not, of course, available for conversion, because there were no funds which the Departments could use to acquire the "rights" of conversion. The Government Departments by their inactivity have settled a question which has during the past few months exercised the minds of trustees generally, and one which has had to be decided by the trustees of most hospitals. The majority of institutions were possessed of holdings of Consols which have now probably been converted into War Loan. In effect this was done by writing down the nominal value of Consols by one-third in exchange for an equal amount of War Loan, yielding 4½%.

One important eventual reason strongly commended this course—viz. that on the issue of further War Loan, yielding a higher rate of interest than 4½%, the right was secured of depositing 4½% War Loan for an equal amount of stock of the future War Loan if issued to return a higher rate of interest than 4½%. A second strong reason was the general opinion that with the removal of the fixed minimum price of Consols—viz. 65—the premier security would fall to 55 or even lower.

On the other hand, trustees in considering the matter have not been unmindful of the fact that Government has the right to repay at par the 4½% Loan on or after December 1, 1925, and in any case the loan must be redeemed in 1945. The opinion was held by sound financiers that Government may find itself able in 1925 or soon after to redeem the existing loan by a loan bearing a lower rate of interest, perhaps 3½%, in which event there would only be a certainty of a 4½% return for ten years. Moreover, if the Government could borrow at 3½%, the value of Consols on this basis would *ipso facto* be about 71¼—and if they could borrow at the still lower rate of 3%, the value of Consols would advance to 83¼. In these circumstances, the position of the converter of Consols would be unfavourable, as he would have parted with Consols for £66 13s. 4d. the value of which might be 71¼ or possibly 83¼.

These and other points had to be considered by holders of Consols. On the whole, there is undoubtedly an inclination to the opinion that the policy of conversion, and of increasing income at the expense of capital, is likely to prove a prudent one, for the removal of the minimum price of Consols has had the effect of reducing the quotation from 65 to 57, a price which may go even lower. Then will be the time for prudent trustees who have converted their Consols to acquire new holdings in England's unredeemable premier security, to yield the attractive return of 5 per cent.

